# Ethanol extract of *Gleditsia sinensis* thorn suppresses angiogenesis *in vitro* and *in vivo*

**DOI:** 10.1186/1472-6882-12-243

**Published:** 2012-12-04

**Authors:** Jin-Mu Yi, Jong-Shik Park, Se-Mi Oh, Jun Lee, Jinhee Kim, Dal-Seok Oh, Ok-Sun Bang, No Soo Kim

**Affiliations:** 1 KM-Based Herbal Drug Research Group, Herbal Medicine Research Division, Korea Institute of Oriental Medicine, 1672 Yuseong-daero, Yuseong-gu, Daejeon 305-811, Republic of Korea

**Keywords:** *Gleditsia sinensis* thorn, Antiangiogenesis, Anticancer, Gene expression, Medicinal herb

## Abstract

**Background:**

*Gleditsia sinensis* thorns have been widely used in traditional Korean medicine for the treatment of several diseases, including obesity, thrombosis, and tumor-related diseases. The aim of the study is to determine the antiangiogenic effect of *Gleditsia sinensis* thorns *in vitro* and *in vivo* in a bid to evaluate its potential as an anticancer drug.

**Methods:**

Ethanol extract of *Gleditsia sinensis* thorns (EEGS) were prepared and used for *in vitro* and *in vivo* assays. *In vitro* antiangiogenic effect of EEGS was determined in HUVEC primary cells by cell migration and tube formation assays. *In vivo* antiangiogenic effect of EEGS was determined by measuring vessel formation and vascular endothelial cells migrating into the implanted matrigels in nude mice. The angiogenesis-related proteins of which expression levels were altered by EEGS were identified by proteomic analysis.

**Results:**

EEGS exerted a dose-dependent antiproliferative effect on HUVEC cells without significant cytotoxicity. Angiogenic properties, such as cell migration and tube formation, were significantly inhibited by EEGS in a dose-dependent manner. New vessel formation was also suppressed by EEGS, as determined by the directed *in vivo* angiogenesis assays in nude mice. EEGS reduced the expression of proangiogenic proteins, endothelin 1 and matrix metallopeptidase 2, in HUVEC cells.

**Conclusions:**

Our findings suggest that EEGS can inhibit angiogenesis by down-regulating proangiogenic proteins, and therefore it should be considered as a potential anticancer drug targeting tumor-derived angiogenesis.

## Background

Angiogenesis is the physiological process of forming new blood vessels from the preexisting vasculature, and it is a vital process during embryonic development. However, in adults angiogenesis is only observed in specific areas, such as the endometrium and ovarian follicle cells [1]. Angiogenesis also plays a key role in many diseases, including cancer, where it promotes tumor growth and metastasis [2]. A continuous supply of nutrients and oxygen is critical for tumor growth; however, these factors are severely limited in the interior of solid tumors, and the tumor core undergoes apoptotic death in the absence of new blood vessels. Moreover, suitable tumor vasculature is also important for removing the metabolic waste produced by tumors, to maintain normal metabolic processes and for tumor development [3]. In fact, the volume of a tumor cannot exceed >1 mm^3^ in an avascular state [4]. Therefore, the inhibition of angiogenesis is a promising strategy for anticancer drug development.

Since 1971, when Folkman hypothesized that tumor growth is dependent on angiogenesis [5], considerable efforts have been dedicated to develop cancer therapies that target angiogenesis. Because angiogenesis is a multi-step and multi-factorial process, each step or factor could be a target of antiangiogenic cancer therapy. Current antiangiogenic therapies include natural angiogenesis inhibitors (e.g., angiostatin), endothelial cell growth inhibitors (e.g., TNP-470), inhibitors of proangiogenic molecules (e.g., vascular endothelial growth factor [VEGF] receptor antibodies), and therapies that interfere with basement membranes and the extracellular matrix (e.g., tissue inhibitors of matrix metallopeptidases [TIMPs]) [2]. Endothelial cells have low mutagenesis rates and are unable to acquire multidrug resistance to cancer therapeutics, making angiogenesis an attractive anticancer target [4]. An additional advantage is the ability of antiangiogenic drugs to target newly forming vessels without harming surrounding normal cells; therefore, they show lower toxicities than traditional cytotoxic chemotherapeutics. Thus cancer patients may be able to receive repeated cycles of therapy without serious side effects [6]. In addition, antiangiogenic cancer drugs have the potential to treat a wide range of solid tumors because most tumors require neovasculature for propagation and metastasis. Previous studies have demonstrated that cancer cells release proangiogenic proteins, such as VEGF [7] and basic fibroblast growth factor (FGF2) [4]. These growth factors recruit endothelial cells and promote their proliferation. Therefore, small molecules that interfere with the proangiogenic signaling pathway are potential anticancer drugs.

Traditional oriental medicine has used various parts of *Gleditsia sinensis* such as thorns, fruits, and anomalous fruits (fruits without seeds) to treat diverse diseases including thrombosis, obesity, and tumor-related disease [8-10]. In oncologic aspect the extract of *Gleditsia sinensis* thorn could prevent colon cancer *in vitro* and *vivo* through the induction of G2/M cell cycle arrest and extracellular signal-regulated kinase 1/2 (ERK1/2) activation [10], and cervical cancer *in vivo* through down-regulation of proliferating cell nuclear antigen (PCNA) and mutant p53 [11]. The extract of anomalous fruits of *Gleditsia sinensis* induced apoptotic cell death in primary leukemic cells of cancer patients [12]. In addition the extract of *Gleditsia sinensis* fruits showed anticancer effects in esophageal squamous cell carcinoma cell lines by inhibiting cyclooxygenase 2 (COX2) expression and telomerase activity [13]. The extract of *Gleditsia sinensis* thorn was also known to have antiatherogenic effect in vascular smooth muscle cells by inhibiting cell proliferation and TNFα-induced matrix metallopeptidase 9 (MMP9) expression [14]. However, the effect of EEGS on angiogenesis and its underlying mechanism are still in question in primary endothelial cells that form blood vessels. In this study, we demonstrated that the EEGS has antiangiogenic potential both *in vitro* and *in vivo*.

The thorn of *Gleditsia sinensis* L. (family Leguminosae) has been widely used in traditional Chinese and Korean medicine for the treatment of several diseases, including obesity, thrombosis, and tumor-related diseases [9,10]. Lee *et al.* demonstrated that ethanol extract of the *Gleditsia sinensis* thorn (EEGS) showed antiproliferative potential in colon cancer cell lines *in vitro* and *in vivo* by inducing cancer cell growth arrest in the G2/M phase [10]. However, the effect of EEGS on angiogenesis and its underlying mechanism are unknown in primary endothelial cells forming blood vessels. In this study, we demonstrated that the EEGS has antiangiogenic potential both *in vitro* and *in vivo*.

## Methods

### Preparation of EEGS

*Gleditsia sinensis* thorns were purchased from Kwangmyungdang Medicinal Herbs (Ulsan, Republic of Korea) in July 2010. Identity of the *Gleditsia sinensis* thorns was confirmed by Dr. Go Ya Choi, Basic Herbal Medicine Research Group, Herbal Medicine Research Division, Korea Institute of Oriental Medicine (KIOM). A voucher specimen (KIOM-CRC-1) was deposited at KM-Based Herbal Drug Research Group, Herbal Medicine Research Division, KIOM, Republic of Korea. Dried *Gleditsia sinensis* thorns (200 g) were finely pulverized and immersed in 70% (v/v) ethanol (100 g/L). The solvent extraction was performed by subjecting the mixtures to two consecutive 1 h periods of ultrasonication. The extracts were filtered through Whatman No.2 filter paper and concentrated in a rotary evaporator. The powdered extract (11.03 g) was homogenized using a mortar and stored at 4°C. The yield of the final extract was approximately 5.52% (w/w).

### Cell culture

Human umbilical vein endothelial cells (HUVEC) were obtained from Lonza (Walkersvill, MD, U.S.A.). They were maintained in EGM-2 endothelial growth medium (Lonza) containing 2% fetal bovine serum (FBS), 0.4% FGF2, 0.1% VEGF, 0.1% R3-insulin-like growth factor 1 (R3-IGF1), 0.1% epidermal growth factor (EGF), 0.04% hydrocortisone, 0.1% ascorbic acid, 0.1% heparin, and 0.1% GA-1000 at 37°C in a humidified atmosphere containing 5% CO_2_. The culture medium was replaced with fresh medium every other day, and the cells were used for experiments between passage number 5 and 10.

### Cell viability

Because the crude ethanol extracts were insoluble in water, EEGS was dissolved in dimethyl sulfoxide (DMSO, Sigma, St Louis, MO, U.S.A.) at a concentration of 20 mg/mL and stored at −70°C until use. One day before drug treatment, 5 × 10^3^ cells were seeded into each well of a 24-well tissue culture plate that contained 450 μL of EGM-2. The cells were then treated with 50 μL of serially diluted test drugs and maintained for various periods. The higher maximum concentration of vehicle (1%) than usual (0.1-0.5%) was used in this study due to low solubility of EEGS. However, we found that neither cell viability nor *in vitro* angiogenesis of HUVEC cells was significantly affected by DMSO up to 1%. Drug-treated HUVEC cells were trypsinized and resuspended in cultured medium. The numbers of total (viable and dead) and dead cells were determined using an ADAM-MC automatic cell counter (NanoEnTek, Seoul, Republic of Korea). In brief, AccuStain T solution is a cell lysis solution supplemented with a DNA staining fluorescent dye (propidium iodide, PI), and AccuStain N solution is a saline solution containing only PI. The numbers of total (viable and dead) and dead cells were counted by mixing an equal volume of cell suspension with AccuStain T and AccuStain N, respectively, and by loading 20 μL of mixed solution into the T and N channels of AccuChip. The number of total cells and the cell viability were automatically calculated by ADAM-MC software.

### Wound healing assay

HUVEC cells, cultured in 24-well plates, were scratched with a yellow tip at 90% confluence and photographs were taken using an inverted microscope (Olympus IX71, Tokyo, Japan). Cells were washed with fresh EGM-2 and further incubated in fresh EGM-2 with various concentrations of EEGS. After 12 h, photographs were taken and wound healing was digitally quantified using MetaMorph image analysis software (Molecular Devices, Downingtown, PA, U.S.A.). The healing area (%) was calculated according to the following formula: healing area (%) = [1-wounded area (t=12 h)/wounded area (t = 0 h)] × 100.

### Tube formation assay

The antiangiogenic potential of EEGS was tested using a Cultrex *in vitro* angiogenesis assay kit (Trevigen, Gaithersburg, MD, U.S.A.) according to the manufacturer’s instructions. One hundred microliters of HUVEC cells (1.5 × 10^5^ cells/mL) were resuspended in EGM-2 with various concentrations of EEGS and added to a 96–well plate that was precoated with basement membrane extracts (BME). After 12 h cultivation at 37°C, tubes were photographed using a microscope. Sulforaphane (5 μM) was included in the tube formation assay as a positive control [15]. The tube length and branch points were digitally quantified using MetaMorph image analysis software.

### *In vivo* angiogenesis assay

*In vivo* angiogenesis was assayed using the directed *in vivo* angiogenesis assay (DIVAA) kit (Trevigen) according to the manufacturer’s instructions. In brief, angioreactors, silicone cylinders closed at one end, were filled with growth factor-reduced BME premixed with combination of angiogenic factors (VEGF, FGF2) and different concentrations of EEGS (0, 50, 100 and 200 μg/ml). Angioreactors were inverted and incubated at 37°C for 1 h to allow gel formation before subcutaneous implantation into the dorsal flanks of the 6-week-old nude mice. After 12 days, the angioreactors were harvested and vessel formation was photographed. Vascular endothelial cells migrating into the BME to form vessels in the angioreactor were quantified by FITC-lectin detection. The fluorescence intensity was measured at a wavelength of excitation 485 nm and emission 510 nm with a fluorescence microplate reader (SPECTRA MAX GEMINI EM, Molecular Devices). The fluorescence intensity is proportional to the number of endothelial cells migrating into the BME gels of implanted cylinder. The relative angiogenesis were normalized to the mean of the positive control. All experiments involving mice were approved by the Institutional Animal Care and Use Committee at the Korea Institute of Oriental Medicine (Protocol # 12–058).

### Proteomic analysis

HUVEC cells were cultured in 100-mm tissue culture dishes containing EGM-2 media until they reached 75-80% confluence. The cells were washed with PBS and incubated in EGM-2 media with or without EEGS for the indicated times. The cultured medium was collected and centrifuged for further analysis. The cells were rinsed with ice cold PBS and solubilized in lysis buffer (1% NP-40, 20 mM Tris–HCl, pH 8.0, 137 mM NaCl, 10% glycerol, 2 mM EDTA, 10 μg/mL aprotinin, 10 μg/mL pepstatin and 10 μg/mL leupeptin) by gentle rocking at 4°C for 30 min. The insoluble debris was removed by centrifugation at 14,000 × g for 5 min at 4°C, and the soluble fractions were collected. Protein concentrations were determined using the BCA protein assay kit (Pierce, Rockford, IL, U.S.A.), and 200 μg of cell lysate or 100 μL of culture supernatant were incubated with human angiogenesis array membranes (Proteome Profiler™, R&D Systems). The proteome profiles were developed according to the manufacturer’s instructions. Membrane-bounds proteins were visualized using Supersignal west femto chemiluminescent substrates (Pierce), and the images were captured on a Fusion SL4 imaging system (Fisher Biotec, Wembley, Australia).

### Immunoassay

The amount of endothelin 1 (EDN1) released from HUVEC cells was quantified using the human EDN1 immunoassay kit (R&D Systems, Minneapolis, MN, U.S.A.) according to the manufacturer's instructions. In brief, cultured medium was centrifuged to remove particulates and the supernatants were aliquoted and stored at −20°C. The recombinant EDN1 standards and samples were loaded into separate wells of a microplate that was precoated with an EDN1 monoclonal antibody, and the samples were incubated for 1 h at room temperature (RT). After washing, EDN1 conjugates were added to each well and incubated for 3 h at RT. After washing, a substrate solution was added and incubated for 30 min. Color development was monitored using a microplate reader (Emax, Molecular Devices) at 450 nm, and the concentration of EDN1 was calculated from the standard curve.

### Activity gel for matrix metallopeptidase 2

The activity of matrix metallopeptidase 2 (MMP2) was determined using a gelatin zymogram as previously described [16]. In brief, cultured medium was centrifuged at 1,000 rpm for 5 min at 4°C to remove cellular debris. The supernatants were then concentrated using a Vivaspin 6 (GE healthcare, Piscataway, NJ, U.S.A.) with a molecular weight cutoff size of 5 kDa. The protein concentration of the medium was quantified using a 2D quant kit (GE healthcare), and 30 μg of protein was mixed with SDS-PAGE loading buffer without reducing agent. The protein samples were incubated at 24°C for 30 min and separated on a 7.5% SDS-PAGE gel copolymerized with 1 mg/mL gelatin (Sigma). The gels were washed three times (30 min at 24°C) with 2.5% (v/v) Triton X-100 to remove SDS, and then incubated in a buffer composed of 50 mM Tris (pH 7.6), 1 μM ZnCl_2_, and 5 mM CaCl_2_ for 18 h at 37°C. The gels were stained with 0.1% Coomassie blue solution.

### Real-time polymerase chain reaction (RT-PCR)

TaqMan RT-PCR was performed to determine the effect of the EEGS on intracellular mRNA levels of EDN1 and MMP2. Total RNA was prepared from HUVEC cells that had been cultured in the presence or absence of EEGS using the Easy-spin™ total RNA extraction kit (iNtRON biotechnology, Seoul, Republic of Korea). The integrity of the isolated total RNA was confirmed by agarose gel electrophoresis. Single-stranded cDNA was synthesized from 5 μg of total RNA using the SuperScript™ III first strand synthesis system (Invitrogen, Carlsbad, CA, U.S.A.). Pre-validated probe and primer sets for EDN1 (ABI ID, Hs00174961_m1; FAM-labeled), MMP2 (ABI ID, Hs01548727_m1; FAM-labeled), and β-actin (ABI ID, Hs99999903_m1; VIC-labeled) were purchased from Applied Biosystems (Foster City, CA, U.S.A.). The PCR reaction and determination of the relative expression of specific genes were carried out in the Applied Biosystems Sequence Detection System 7500.

### Statistics

The differences in continuous variables were determined by a one-way analysis of variance (ANOVA) and Tukey’s HSD post-hoc test. Statistical significance was set at *p*<0.05.

## Results

### EEGS inhibits cell proliferation but does not induce cell death in HUVEC cells

**Figure 1:**
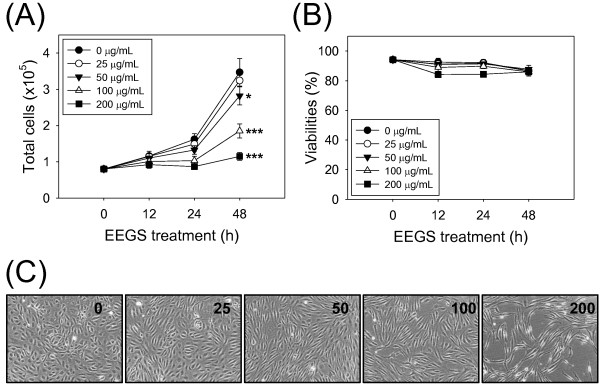
**Antiproliferative effect of EEGS in HUVEC cells. **Exponentially growing HUVEC cells were treated with various concentrations (0–200 μg/mL) of EEGS for the indicated time periods. The total cell number (**A**) and viability (**B**) were determined as described in the Methods. The effect of EEGS on HUVEC growth and morphological changes were observed under the microscope at 200 x magnification (**C**). The data are presented as the mean±S.D. of at least three independent experiments. Statistical analysis was performed at the 48 h time point. *p<0.05 and ***p<0.001 compared with control treatment.

Proliferation of endothelial cells in response to angiogenic stimuli is a critical step during new vessel formation [17]. To determine the effect of EEGS on HUVEC cell growth, HUVEC cells were cultured in EGM-2 media containing endothelial growth factors and exposed to increasing concentrations of EEGS (0–200 μg/mL). At regular time intervals, total (viable and dead) cell numbers and viability were calculated using an automatic cell counter that assessed the cytoplasmic membrane integrity as described in the Methods. As shown in Figure 1A, EEGS treatment inhibited cell proliferation in a dose-dependent manner. A significant inhibitory effect on cell proliferation was observed in response to EEGS at ≥50 μg/mL concentrations. The highest dose (200 μg/mL) of EEGS inhibited cellular proliferation completely throughout the 48 h cultivation period. However, the antiproliferative effect of EEGS was not related to cytotoxicity (Figure 1B). Although a slight decrease in cell viability was observed in the EEGS-treated HUVEC cells, no significant cytotoxicity was observed at doses of up to 200 μg/mL EEGS during the 48 h cultivation period. The antiproliferative potential of EEGS was also observed under the microscope (Figure 1C). Although increasing concentrations of EEGS induced morphological changes and growth arrest, significant cell death was not observed during 48 h of treatment.

### EEGS inhibits angiogenic properties in HUVEC cells *in vitro*

**Figure 2:**
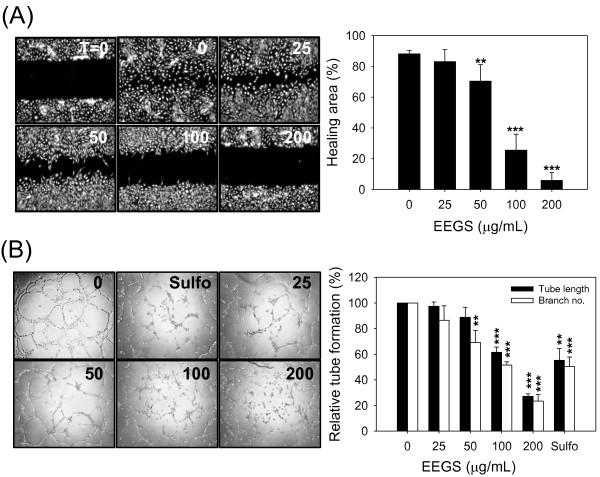
**Inhibitory effect of EEGS on angiogenic properties in HUVEC cells. **(**A**) HUVEC cells at 90% confluence were scratched with pipette tips and cultured with various concentrations (0–200 μg/mL) of EEGS for 12 h. The healing areas covered by migrated cells (left) was calculated using image analysis software (right). The data are presented as percentages of the healed area compared to the initial scratched area (t = 0). (**B**) HUVEC cells were plated in a 96-well plate that was precoated with BME, and the cells were subsequently treated with various concentrations (0–200 μg/mL) of EEGS. After 12 h, the tubes were photographed under the microscope at 100 x magnification (left). Tube length and branch numbers were measured using image analysis software (right). Sulforaphane (Sulfo, 5 μM) was used as a positive control for inhibition of tube formation. The data are presented as relative means compared with control treatment. Data are presented as the mean±S.D. of at least three independent experiments. **p<0.01, and ***p<0.001 compared with control treatment.

Angiogenesis mediated by vascular endothelial cells is characterized by cell migration to designated sites of new blood vessel formation. Thus, the antiangiogenic potential of EEGS in HUVEC cells was determined using an *in vitro* wound healing assay. In the absence of EEGS treatment, HUVEC cells found at the border of scratches (Figure 2A, left panel, T = 0) migrated rapidly toward the wounded area and covered it within 12 h (0 μg/mL of EEGS). However, cell mobility was slowed by EEGS treatment in a dose-dependent manner, and HUVEC cells treated with 200 μg/mL of EEGS showed no movement. A significant decrease in cell mobility was observed at a concentration of ≥50 μg/mL, and only 10% of the wound was covered at an EEGS concentration of 200 μg/mL (Figure 2A, right panel). In addition, we evaluated the effect of EEGS treatment on tube formation by HUVEC cells. On a matrix-coated surface, HUVEC cells are capable of building tubes via connecting to neighboring cells in the presence of growth factors (Figure 2B, left panel, 0 μg/mL of EEGS). However, in the presence of EEGS, the intercellular connection was limited and HUVEC cells failed to form tubes. To quantify the degree of tube formation, photographs were taken at the end of each experiment under the microscope, and tube length and branch numbers were calculated using image analysis software (Figure 2B, right panel). Both parameters decreased in a dose-dependent manner following EEGS treatment. Statistical analyses revealed that both tube length and branch number were significantly reduced at ≥100 μg/mL concentrations of EEGS. The inhibitory effect of 100 μg/mL of EEGS on HUVEC cell tube formation was comparable to that of 5 μM sulforaphane, a positive control for tube formation inhibition.

### EEGS inhibits angiogenesis *in vivo*

**Figure 3:**
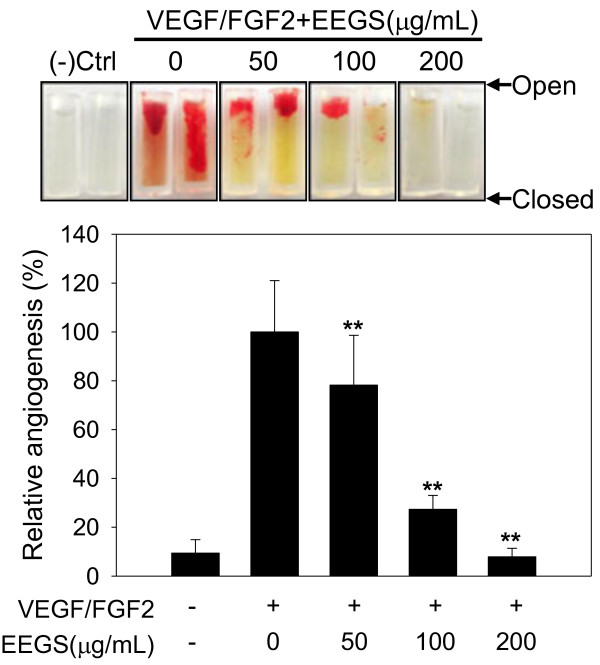
**The antiangiogenic effects of EEGS *in vivo.*** Angioreactors filled with BME premixed with combination of VEGF/FGF2 angiogenic factors and increasing concentrations of EEGS (0–200 μg/mL) were implanted into the dorsal flank of nude mice. After 12 days, the implanted cylinders were harvested (upper), and vascular endothelial cells migrated into the BME gels of bioreactors were quantified by FITC-lectin detection (lower). Negative control represents the bioreactors without VEGF/FGF2 angiogenic factors. The relative angiogenesis (%) were normalized to the mean of the VEGF/FGF2-treated positive control. Data are presented as the mean±S.D. of four cylinders per group. **p<0.01 compared with positive control.

Because EEGS inhibited *in vitro* angiogenic properties, such as cell migration and tube formation, we next investigated its effect on angiogenesis *in vivo* using commercially available direct *in vivo* angiogenesis kit as described in Methods. As shown in Figure 3 (top panel), massive vessel ingrowth from the open ends of angioreactors was observed in the presence of VEGF/FGF2 angiogenic factors but in the absence of EEGS (positive control). However, EEGS treatment suppressed the vessel ingrowth induced by angiogenic factors in a dose-dependent manner as determined by FITC-lectin labeling of endothelial cells to form new vessels (bottom panel). Antiangiogenic effect of EEGS was observed at ≥50 μg/mL concentration and complete inhibition of vessel formation was observed at 200 μg/mL of EEGS. New vessel formation was limited in the absence of both VEGF/FGF2 and EEGS (negative control).

### EEGS down-regulates the expression of proangiogenic proteins

**Figure 4:**
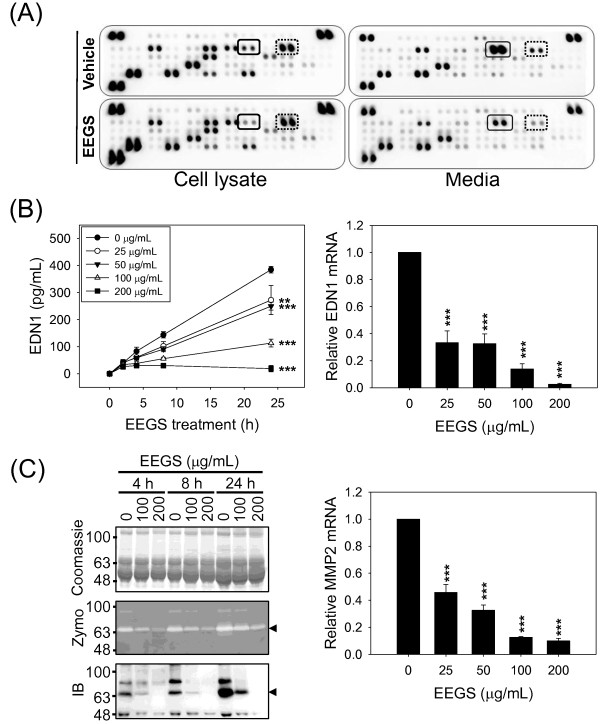
**Down-regulation of proangiogenic proteins by EEGS in HUVEC cells. **(**A**) HUVEC cells were incubated in the presence (100 μg/ml) or absence of EEGS for 8 h. The protein expression profiles in the cultured medium and cell lysates were analyzed using a human angiogenesis array kit. The closed and dotted rectangles represent the duplicated EDN1 and FGF2 spots in the proteome membrane chips, respectively. (**B**) HUVEC cells were treated with increasing concentrations (0–200 μg/mL) of EEGS for 24 h. The amount of EDN1 released into the cultured medium after EEGS treatment was quantified by immunoassay (left). Changes in EDN1 mRNA expression levels were determined by RT-PCR (right). Total RNAs and cDNAs were prepared from HUVEC cells that were treated with increasing concentrations of concentrations of EEGS for 8 h. (**C**) Changes in MMP2 activity in the HUVEC cultured medium were measured by gelatinolytic zymogram analysis (left). MMP2 activity was observed at the expected molecular weight (67 kDa) on the gelatin-incorporated SDS-PAGE gel. Coomassie staining of a SDS-PAGE gel without gelatin confirmed equal protein loading. The MMP2 active bands were confirmed by western blot analysis. The effect of EEGS on the levels of intracellular MMP2 mRNA was determined by RT-PCR (right). Total RNAs and cDNAs were prepared from HUVEC cells that had been treated with increasing concentrations of EEGS for 8 h. Data are presented as the mean±S.D. of at least three independent experiments. **p<0.01 and ***p<0.001 compared with control treatment.

To identify proteins of which expression levels are altered in response to EEGS treatment, a proteomic analysis was carried out using cultured media or cell lysates prepared from HUVEC cells that had been treated with 100 μg/mL EEGS or vehicle for 8 h. Membranes arrayed with specific antibodies for angiogenesis-related proteins were incubated with cultured medium or cell lysates, and the protein expression profiles were compared by analyzing dot intensities (Figure 4A). We observed significant changes in the protein expression of FGF2 and EDN1. Treatment of 100 μg/mL EEGS decreased the EDN1 expression by 34.8% (*p*<0.001) in the cultured media, and by 55.2% (*p*<0.001) in the cell lysate compared with the negative vehicle control. Because the expression of FGF2, which was supplemented in EGM-2 endothelial growth medium, was decreased by EEGS only in cultured media (41.7%, *p*<0.01) but not in the cell lysate (122.5%, *p*=0.210), it was excluded from our further studies. It is possible that HUVEC cells treated with EEGS may consume FGF2 more rapidly than HUVEC cells treated with DMSO by an unknown mechanism. The proangiogenic protein, EDN1, showed the most dramatic and reproducible change in protein levels in both cell lysate and cultured media. To verify the EEGS-induced EDN1 down-regulation observed in the proteomic analysis, we performed an EDN1 immunoassay using a commercially available kit (Figure 4B, left panel). Without EEGS treatment, extracellular EDN1 increased continuously throughout the culture period. However, in the presence of EEGS, the rate of EDN1 production decreased in a dose-dependent manner. Treatment with 200 μg/mL EEGS almost completely inhibited EDN1 production after 4 h. Next, we determined the effect of EEGS treatment on the level of intracellular EDN1 mRNA using RT-PCR. Total RNA and cDNA were prepared from HUVEC cells that were treated with increasing concentrations (0–200 μg/mL) of EEGS for 8 h. EEGS treatment reduced the level of intracellular EDN1 mRNA in a dose-dependent manner (Figure 4B, right panel), and 200 μg/mL of EEGS decreased EDN1 mRNA levels by more than 90%. These data suggest that EEGS down-regulates EDN1 mRNA levels, leading to reduced EDN1 production.

MMP2 and MMP9 are key enzymes involved in angiogenesis during cancer development. Proteomic analysis revealed no change in MMP9 expression following EEGS treatment. However, MMP2 was not arrayed on the proteome membrane chip, and therefore we investigated the effect of EEGS on the activity of MMP2 in HUVEC cell cultured media using zymogram. MMP2 can hydrolyze a gelatin substrate incorporated into an SDS-PAGE gel, and gelatin hydrolysis by MMP2 can be visualized by Coomassie staining. The activity of MMP2 in the zymogram was decreased by EEGS in a dose-dependent manner (Figure 4C, left panel). Western blot analysis revealed that reduced MMP2 activity correlated with a decrease in the level of MMP2 protein secreted into the cultured medium. To further understand the EEGS-induced reduction in MMP2 activity, the intracellular mRNA levels of MMP2 were investigated using RT-PCR. Total RNA and cDNA were prepared from HUVEC cells that were treated with increasing concentrations (0–200 μg/mL) of EEGS for 8 h. Intracellular mRNA levels of MMP2 were reduced in a dose-dependent manner (Figure 4C, right panel), which may explain the decrease in MMP2 activity in cultured media, as shown in the zymogram.

## Discussion

Although massive tumors can be treated by surgical intervention, treatment of small primary tumors or cancers undergoing metastasis largely relies on chemotherapy [4]. However, the clinical use of cytotoxic cancer drugs, which target uncontrolled, dividing cancer cells, is limited by the severe side effects caused by killing fast dividing normal cells, such as blood cells. Therefore, many cytotoxic cancer drugs currently in development are designed to attack only cancer cells. This targeted therapy is achieved by conjugating cytotoxic drugs with monoclonal antibodies that recognize cancer specific molecules. However, a disadvantage of these “magic bullet” cancer drugs is that they demonstrate variable efficacy depending on the cancer type and the genetic background of the patient [18,19]. Another limitation of cytotoxic anticancer drugs is that these chemicals can induce drug resistance in cancer cells as a result of tumor drug efflux [20]. Therefore, the drug dose may need to be increased to induce the same therapeutic response over time.

New blood vessel formation, or angiogenesis, is important for many physiological processes throughout the entire human lifespan, including fetal development. It is also critical for tumor development, as first suggested almost 40 years ago by Folkman [5,21]. Anticancer drugs developed to inhibit angiogenesis have been demonstrated to inhibit tumor growth by targeting molecules within the angiogenic signaling pathway. Bevacizumab (trade name, AVASTIN®, Genentech/Roche), a monoclonal antibody raised against human vascular endothelial growth factor A (VEGFA) inhibits the VEGFA signaling pathway, which is involved in tumor-derived angiogenesis [22]. It was the first FDA-approved drug for the treatment of metastatic colon cancer and advanced non-small cell lung cancer [23,24]. Because of their relatively low cytotoxicity, antiangiogenic drugs may be used repeatedly and for long-term cancer therapy. Tumor-driven angiogenesis is a nearly universal characteristic of cancer, suggesting that antiangiogenic drugs may have a wide range of clinical applications for cancer therapy [25]. Three major strategies that have been explored so far are as follows: 1) blockade of pro-angiogenic growth factors and their specific signaling pathways, 2) enhancement of the levels of antiangiogenic factors, and 3) disruption of abnormal cancerous vascular function [25]. The aim of this study was to validate EEGS as a potential antiangiogenic agent because the source of EEGS, the *Gleditsia sinensis* thorn, is a herbal drug that has been commonly prescribed for thrombosis and tumor-related diseases in the traditional Korean medicine. The antiangiogenic potential of EEGS was explored using well established *in vitro*/*in vivo* angiogenesis assays.

Angiogenesis is a multi-step process requiring coordinated endothelial functions, such as cell migration, proliferation and extracellular matrix remodeling [16]. In this study, we employed HUVEC cells for *in vitro* assays representing several angiogenic functions because they are a well-known macrovasculature model and are susceptible to antiangiogenic drugs [26]. Our *in vitro* assays demonstrated that EEGS inhibited HUVEC cell proliferation (Figure 1A) and cellular mobility (Figure 2A). These cellular processes are related to the angiogenic properties of HUVEC cells, and inhibition of these angiogenic properties by EEGS resulted in global inhibition of HUVEC cell tube formation on matrigel at EEGS concentrations of ≥50 μg/mL for tube branching and ≥100 μg/mL for both tube branching and length (Figure 2B). EEGS-mediated inhibition of the angiogenic functions of HUVEC cells was replicated in the direct *in vivo* angiogeneis assays using nude mice (Figure 3).

Cell growth, which is determined by the balance between cell amplification and death, affects angiogenesis. To rule out the effect of EEGS-induced cytotoxicity on angiogenesis, we investigated the cytotoxicity of EEGS in HUVEC cells in the presence of increasing concentrations of drug, up to 200 μg/mL. Although EEGS induced dramatic antiproliferative effects at a concentration of ≥50 μg/mL (Figure 1A), cell viabilities was not significantly changed at doses as high as 200 μg/mL, as determined by membrane integrity or morphological observation (Figure 1B and C). These results indicate that the antiangiogenic potential of EEGS is not due to cytotoxicity. Therefore, we can separate the antiangiogenic effects of EEGS from its cytotoxic effect within the range of concentrations used in this study (0–200 μg/mL). Paclitaxel, a microtubule-damaging anticancer agent, exerts similar *in vitro* effects on endothelial cells. At high concentrations, paclitaxel exerts cytotoxic effects by inducing mitochondria-mediated apoptosis; however, at low concentrations, it induces cytostatic effects by slowing down the cell cycle, which is associated with antiangiogenic activity in endothelial cells [26]. The inhibitory effect of EEGS on angiogenic functions in HUVEC cells appeared to be reversible because removal of EEGS was sufficient to release cells from EEGS-induced cell migration arrest (data not shown). Recently, Lu *et al.* (2012) [27] reported that six active components of saponin fraction from *Gleditsia sinensis* fruits (gleditsiosides B, I, J, O, Q, and B) exerted *in vitro* antiangiogenic effects on HUVEC cells. However, they also showed that the active saponin compounds induced significant apoptotic cell death in HUVEC cells by enhancing caspase 3/8 expression, which were quite different results from ours. Therefore, our data suggest that the antiangiogenic potential of EEGS may come from active constituent(s) other than saponin compounds.

Proteomic and immunoassay studies showed that both intracellular and extracellular levels of EDN1 were decreased by EEGS treatment in a dose-dependent manner (Figure 4A and B). Furthermore, intracellular EDN1 mRNA levels correlated with changes in extracellular EDN1 levels. EDN1 is one of three 21-amino acid peptide family members (EDN1, EDN2, and EDN3) [28], and it is known to be a proangiogenic modulator that promotes endothelial cell proliferation and migration through two G protein coupled receptors (ET_A_R and ET_B_R) [16]. EDN1 is also known to have effects on the growth and progression of various tumor types by affecting proliferation and resistance to apoptosis [28]. In our study, EEGS treatment reduced the extracellular and intracellular levels of EDN1, suggesting that reducing EDN1 is one of the mechanisms by which EEGS inhibits the angiogenic functions of HUVEC cells.

Because some effective antitumor agents targeting tumor vessel formation have been shown to reduce MMP2 activity [29,30] and MMP2 produced by endothelial cells is known to contribute to the progress of angiogenesis [17], we investigated MMP2 enzyme activity in response to EEGS treatment. The activity of extracellular MMP2 was decreased by EEGS treatment in a dose-dependent manner (Figure 4C). Each regulatory step, such as transcription, translation, translational modification or extracellular secretion of expressed proteins can affect MMP2 activity in cultured media. In the present study, we observed that the decrease in MMP2 activity in HUVEC cell cultured media correlated with decreased levels of extracellular MMP2 proteins (Figure 4C, left panel) and intracellular MMP2 mRNA (Figure 4C, right panel). Therefore, EEGS down-regulates MMP2 mRNA expression, leading to reduced MMP2 protein. However, we did not investigate the mechanism by which EEGS treatment down-regulates MMP2 mRNA expression in the present study. It is possible that EEGS inhibits the transcription of MMP2 or affects the stability of MMP2 mRNA in HUVEC cells. Taken together, our data suggest that EEGS exerts its antiangiogenic effects on HUVEC primary endothelial cells by inhibiting the expression and/or activity of proangiogenic proteins.

Most previous phytochemical studies on *Gleditsia sinensis* were carried out using its fruit and anomalous fruit parts. The single compounds from the fruits or anomalous fruits of *Gleditsia sinensis* have been isolated as triterpene (echinocystic acid), flavonoid (aromadendrin), polyphenol (ellagic acid glycosides), and triterpenoid saponins (gleditsioside A-K, N-Q, and Z) [31-40]. Their identified pharmacological activities were antagonistic against dopamine D1 receptor (gleditsioside F) [33], protective against acute myocardial ischemia (echinocystic acid) [32] or type 2 diabetes mellitus (aromadendrin) [35], antiallergic in mast cells (saponins) [41], and cytotoxic to leukemic cells (gleditsioside E) [38]. The single compounds from the *Gleditsia sinensis* thorns were isolated as a lupane acid with anti-HIV activity [42], and triterpenoid (D:C-friedous-7-en-3-one) and sterols with antimutagenic activity [43]. To our knowledge, there was no study reporting antiangiogenic active compound(s) from the *Gleditsia sinensis*. We are trying to identify antiangiogenic active single compound(s) from the extract of *Gleditsia sinensis* thorn using *in vitro* activity-guided fractionation.

## Conclusions

Because tumor angiogenesis is a very complex progress involving diverse cell types, agents with multi-targets or a combination of targeted single agents are needed to target angiogenesis as an anticancer strategy. *In vitro* and *in vivo* assays clearly identified EEGS as an antiangiogenic herbal drug. Thus, EEGS is a promising antiangiogenic cancer drug that warrants further development. The inhibitory effects of EEGS are likely due to down-regulation of intra/extracellular proangiogenic modulators, such as EDN1 and MMP2 enzymes. At present, the mechanisms by which EEGS suppresses the expression of proangiogenic modulators were not fully characterized in this study. Further studies are needed to identify the molecule(s) responsible for EEGS-mediated antiangiogenesis.

## Competing interests

The authors have declared that there is no competing interest.

## Authors' contributions

JMY and NSK carried out study concept, experimental design, data acquisition and analysis, and drafted the manuscript. JSP and SMO carried out data acquisition and analysis, and assisted with drafting manuscript. JL, JK, and DSO assisted with study concept, drafting, and revising the manuscript. OSB supervised the study and assisted with study concept and revising the manuscript. All authors read and approved the final manuscript for submission.

## Pre-publication history

The pre-publication history for this paper can be accessed here:

http://www.biomedcentral.com/1472-6882/12/243/prepub
